# Clinical Impact of Cardiac Fibrosis on Arrhythmia Recurrence after Ablation in Adults with Congenital Heart Disease

**DOI:** 10.3390/jcdd10040168

**Published:** 2023-04-13

**Authors:** Francesco Perna, Alessandro Telesca, Roberto Scacciavillani, Maria Lucia Narducci, Gianluigi Bencardino, Gaetano Pinnacchio, Francesco Raffaele Spera, Rocco Sabarese, Gianluca Comerci, Gemma Pelargonio

**Affiliations:** 1 Cardiac Arrhythmia Unit, Fondazione Policlinico Universitario Agostino Gemelli IRCCS, Largo Agostino Gemelli 8, 00168 Rome, Italy; francesco.perna@policlinicogemelli.it (F.P.);; 2 Department of Cardiovascular Sciences, Catholic University of the Sacred Heart, Largo Francesco Vito 1, 00168 Rome, Italy

**Keywords:** adult with congenital heart disease, catheter ablation, fibrosis, arrhythmias, electroanatomical mapping

## Abstract

Background. Adults with congenital heart disease (ACHD) are often affected by cardiac arrhythmias requiring catheter ablation. Catheter ablation in this setting represents the treatment of choice but is flawed by frequent recurrencies. Predictors of arrhythmia relapse have been identified, but the role of cardiac fibrosis in this setting has not been investigated. The aim of this study was to determine the role of the extension of cardiac fibrosis, detected by electroanatomical mapping, in predicting arrhythmia recurrencies after ablation in ACHD. Materials and Methods. Consecutive patients with congenital heart disease and atrial or ventricular arrhythmias undergoing catheter ablation were enrolled. An electroanatomical bipolar voltage map was performed during sinus rhythm in each patient and bipolar scar was assessed according to the current literature data. During follow-up, arrhythmia recurrences were recorded. The relationship between the extent of myocardial fibrosis and arrhythmia recurrence was assessed. Results. Twenty patients underwent successful catheter ablation of atrial (14) or ventricular (6) arrhythmias, with no inducible arrhythmia at the end of the procedure. During a median follow-up period of 207 weeks (IQR 80 weeks), eight patients (40%; five atrial and three ventricular arrhythmias) had arrhythmia recurrence. Of the five patients undergoing a second ablation, four showed a new reentrant circuit, while one patient had a conduction gap across a previous ablation line. The extension of the bipolar scar area (HR 1.049, CI 1.011–1.089, *p* = 0.011) and the presence of a bipolar scar area >20 cm^2^ (HR 6.101, CI 1.147–32.442, *p* = 0.034) were identified as predictors of arrhythmia relapse. Conclusion. The extension of the bipolar scar area and the presence of a bipolar scar area >20 cm^2^ can predict arrhythmia relapse in ACHD undergoing catheter ablation of atrial and ventricular arrhythmias. Recurrent arrhythmias are often caused by circuits other than those previously ablated.

## 1. Introduction

Adults with congenital heart disease (ACHD) represent nowadays a growing population due to advancements in surgery, interventional cardiology, and pharmacological therapy, which allows them to survive to older ages in developed counties. In the USA, ACHD are estimated to be about 1.4 million, with a prevalence of up to 6.5 per 1000, outnumbering children nearly 1–1.5-fold [1,2]. This has led physicians to face a population that includes individuals with complex structural abnormalities, impaired hemodynamics, myocardial fibrosis, and multiple surgical scars, bearing a high risk for major cardiovascular complications and a natural predisposition toward recurrent cardiac arrhythmias, most often represented by intra-atrial reentrant tachycardia (IART) [3,4].

Arrhythmias usually represent a main concern in ACHD, because they can severely burden the quality of life and impair hemodynamic stability in patients who are already barely compensated while in sinus rhythm. Moreover, antiarrhythmic drugs are often ineffective or contraindicated in most ACHD. Class IC drugs can be proarrhythmic in patients with extensive fibrosis or conduction disturbances and decrease survival in patients with ventricular dysfunction [5]. Amiodarone is highly effective in preventing arrhythmia recurrence, but side effects and poor tolerability usually lead to discontinuation, especially in relatively young populations [6]. 

Radiofrequency (RF) ablation is a well-established therapeutic approach for arrhythmias in ACHD, with a rate of acute arrhythmia termination as high as 96% and long-term arrhythmia-free survival rate of 46–89% [7,8,9]. The presence of slow conduction areas and potentially identifiable isthmuses, due to myocardial fibrosis and surgical scars, make catheter ablation particularly attractive as a first-line treatment in the era of high-density electroanatomical mapping (EAM) and multi-electrode mapping catheters [10,11].

Several groups have investigated RF ablation in the treatment of arrhythmias in ACHD, reporting few data on long-term follow-ups and predictors of recurrences [7,8,12,13]. To date, the role of atrial fibrosis in predicting success after catheter ablation has not been explored in detail.

The aim of this study was to assess the role of fibrosis, identified by high-density electroanatomical mapping, as a predictor of arrhythmia recurrence after catheter ablation of arrhythmias in ACHD in a single referral center. 

## 2. Material and Methods

### 2.1. Patients’ Characteristics

In this observational study, twenty consecutive patients with ACHD and atrial or ventricular tachyarrhythmias undergoing catheter ablation at our institution from July 2012 to April 2022 were prospectively enrolled. Procedures performed before July 2012 were not considered in order to prevent confounding factors related to technology advancements. All the enrolled patients were aged between 18 and 65 years and suffered from symptomatic atrial or ventricular tachycardias with an indication toward catheter ablation according to the current guidelines [14]. 

For each patient, clinical information was collected before the ablation procedure, including anthropometric data (age, gender, and body mass index); cardiac risk factors and cardiovascular history; type and complexity of the congenital disease and surgical correction; type of arrhythmia; left ventricular ejection fraction (LVEF) or systemic ventricle ejection fraction (EF); New York Heart Association (NYHA) functional class; and pharmacological therapy.

### 2.2. Electrophysiology Procedure (Electroanatomical Mapping and Catheter Ablation)

All the procedures were performed under local anesthesia and conscious sedation with intravenous midazolam and/or pethidine in the electrophysiology lab. Antiarrhythmic drugs were discontinued five half-lives before the intervention. In patients presenting in tachycardia, termination of the arrhythmia was obtained with either overdrive pacing or electrical cardioversion. During sinus rhythm, an endocardial high-density electroanatomical voltage map was performed using a multi-electrode mapping catheter (Intella NAV Orion, Boston Scientific, Cambridge, MA, USA; PentaRay, Biosense Webster, Inc, Diamond Bar, CA, USA; HD Grid, Abbott, Abbott, North Chicago, IL, USA) with one of three electroanatomical mapping systems, depending on the operator’s preference: Rhythmia (Boston Scientific, Cambridge, MA, USA), CARTO 3 (Biosense Webster, Inc, Diamond Bar, CA, USA), or EnSite NavX (Abbott, North Chicago, IL, USA). Based on voltage thresholds depending on the chamber of interest, areas of myocardial scars, healthy areas, and border zones were identified. In particular, for bipolar mapping, according to the literature data, an atrial scar was defined as areas with a voltage < 0.2 mV; low voltage areas (border zone) had a voltage between 0.2 and 0.5 mV; areas with a bipolar voltage > 0.5 mV were considered as healthy areas [15,16,17]. Ventricular areas with a bipolar voltage < 0.5 mV were considered as a scar, while areas with a voltage between 0.5 and 1.5 mV were defined as low voltage areas (border zone) and areas with a voltage > 1.5 mV were considered a normal myocardium [18]. At unipolar mapping, a ventricular scar was identified as an area with a voltage < 5.5 mV [19]. Total extension of the scar areas was expressed as both the absolute area in cm^2^ and percentage of the mapped area for the chamber of interest. Intracardiac bipolar electrograms (EGMs) were recorded by a computerized electrophysiological recording system. For all patients, complete substrate maps of the chamber of interest were obtained. After performing voltage mapping, a programmed atrial or ventricular stimulation (depending on the clinical arrhythmia) was performed from the high right atrium (HRA), coronary sinus (CS), right ventricular apex (RVA), or right ventricular outflow tract (RVOT) to induce the clinical arrhythmia. If the arrhythmia was hemodynamically tolerated, activation mapping and entrainment maneuvers were performed to understand the mechanism of the tachyarrhythmia and to identify the site of origin of a focal tachycardia or a critical isthmus of a reentrant tachycardia as possible targets for ablation. After EAM, radiofrequency ablation (RFA) of the target sites was performed. In patients with a reentrant arrhythmia, we aimed at connecting adjacent scars or electrically silent structures (e.g., vena cava and tricuspid valve) to achieve a conduction block across the line, as verified by electrophysiological maneuvers. In patients whose activation map showed a focal pattern, the zone of earliest activation was targeted for ablation. After termination of the arrhythmia, reinduction was attempted using the standard stimulation protocols and isoprenaline administration. Acute procedural success was defined by termination of the clinical arrhythmia, non-inducibility with complete stimulation protocols, and for reentrant arrhythmias, a bidirectional block across the ablation line proven by pacing along both sides. At the end of the intervention, an echocardiographic examination was performed to rule out the presence of pericardial effusion, and the introducer sheaths were removed. Patients were then transferred to the cardiology ward for multiparametric monitoring.

### 2.3. Follow-Up and Endpoint Definition

In the absence of arrhythmic events during telemetry monitoring or complications, all patients were discharged the day after the procedure and followed up in our Outpatient Clinic at 1, 3, 6, and 12 months after ablation and then at 6-month intervals. Patients were assessed for any relapse of tachyarrhythmias or symptoms, and electrocardiogram (ECG) and 24-h Holter ECG recording were performed. The study endpoint consisted of arrhythmic relapse during the follow-up period, documented by any ECG modality (standard 12-lead ECG, Holter ECG, or implantable device interrogation). We analyzed the correlation between the presence and the extent of the bipolar scar and arrhythmia recurrence after ablation.

### 2.4. Statistical Analysis

Continuous variables are expressed as the mean ± standard deviation or median ± interquartile ranges for non-normally distributed variables. Categorical differences between groups were examined using Pearson’s chi-square test, while the unpaired Student’s *t*-test or the Mann–Whitney *U* test for non-normally distributed variables were used to compare differences between means. Given the sample size and event rates, the univariate Cox proportional hazards regression analysis together with Kaplan–Meier curves were performed to assess the association of baseline clinical variables with the endpoint of arrhythmia relapse. The variables were included in the analysis with regards to their possible association with arrhythmic events. The results are presented as hazard ratios (HRs) and 95% confidence intervals (CI). A *p*-value < 0.05 was considered significant for all statistical determinations. All analyses were performed using SPSS Statistics 23 software for Windows.

## 3. Results

### 3.1. Patients’ Characteristics

Twenty consecutive patients (13 men, 65%) with CHD and a diagnosis of atrial or ventricular tachyarrhythmia were enrolled in our observational study. Fourteen patients (70%) were treated for symptomatic atrial arrhythmia and six patients (30%) for a symptomatic ventricular arrhythmia. None of them had a history of previous endocardial ablation. The mean age at the time of their first ablation was 39 ± 10 years (24–64 years). Five patients (25%) had a simple congenital heart disease (CHD), while fifteen patients (75%) had a complex CHD. Tetralogy of Fallot (TOF) was the most frequent CHD (nine patients), followed by atrial septum defect (ASD, five patients, one of them with an associated partial atrioventricular canal defect (ACD) and another with truncus arteriosus), transposition of great arteries (TGA, two patients, one of these with associated situs viscerum inversus, ostium secundum ASD, and VSD) and aortic coartaction (two patients, one of these with complete ACD and a bicuspid aortic valve). The remaining two patients had ventricular septum defects with tricuspid valve atresia and Ebstein’s anomaly, respectively. All patients had a surgically repaired CHD, except for the case of Ebstein’s anomaly. The type of CHD and surgical correction of each patient are listed in Table 1.

Most patients (17, 85%) were in NYHA functional class ≤ II, while three patients were in NYHA class III. The mean LVEF was 52.6 ± 9.7% (24–69%). Three patients had severely impaired LV systolic function with systemic ventricular EF < 30%. The most represented cardiovascular risk factor present in our study population was arterial hypertension (five patients, 25%). All the patients were symptomatic at presentation, while 18 (90%) of them remained symptomatic despite antiarrhythmic therapy before ablation; 12 patients (60%) were treated with beta-blockers, 5 patients (25%) with amiodarone, 3 patients (15%) with class Ic antiarrhythmic drugs (AADs), and 2 patients (10%) with sotalol. Two patients (10%) were not on AADs because of sinus node disease. Six patients (30%) were on loop diuretics, and five patients (25%) were on angiotensin converter enzyme inhibitors (ACE-Is) or angiotensin receptor blockers (ARBs).

Baseline characteristics of the patients’ population are described in Table 2.

### 3.2. Electroanatomical Mapping and Catheter Ablation

**Figure 1:**
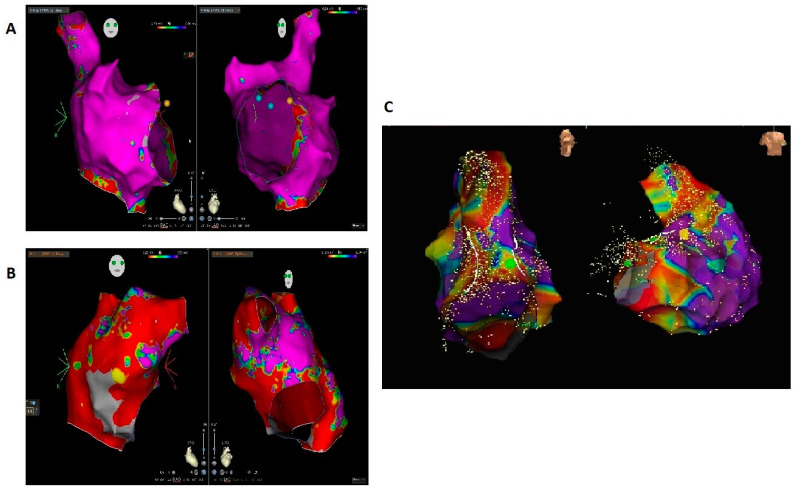
(**A**) Right atrial bipolar map of a patient almost without a scar, which did not show arrhythmia relapse of isthmus-dependent atrial flutter during follow-up. (**B**) Right atrial bipolar map of a patient with intra-atrial reentrant tachycardia and an extensive scar, who had arrhythmia recurrence. (**C**) Right ventricular bipolar map of a patient with ventricular tachycardia showing an extensive scar; the patient had arrhythmia recurrence during follow-up.

Electroanatomical voltage mapping was performed with Rhythmia Mapping System in 14 patients (70%), with CARTO3 system in 4 patients (20%), and EnSite NavX (Abbott) in 2 patients (10%). The mean number of collected mapping points was 8774 ± 6051 (341–22934), while the mean total mapped area was 167.5 ± 103 cm^2^ (34–687 cm^2^). For bipolar mapping, all patients had at least one area of electroanatomic scar tissue (voltage < 0.2 mV in the atria or < 0.5 mV in the ventricles), and the mean total bipolar scar area was 27.2 ± 15.2 cm^2^ (5.6–103.5 cm^2^) (Figure 1). Ten patients (50%) had a total scar area > 20 cm^2^ at the bipolar substrate mapping. The mean ratio of bipolar scar tissue over the total mapped area was 22.6% ± 12%. Five out of six patients with ventricular tachyarrhythmias had unipolar scar areas.

Reentrant tachycardia was the main mechanism encountered (Table 2). Twelve patients (60%) had a right-sided intra-atrial reentrant tachycardia, with six of them (30% of the total population) involving the cavo-tricuspid isthmus. The arrhythmia circuit was in the left atrium in two patients (10%). A bi-atrial map was acquired in only one case (5%) due to the induction of a left atrial flutter during programmed atrial stimulation following successful ablation of the clinical right atrial tachycardia. A total of six patients (30%) presented with ventricular tachycardia. Five of them had a surgically corrected TOF and right-sided ventricular reentrant circuit (four) or focal origin (one); pulmonary infundibulum or the right ventricular outflow tract were involved in all cases. One patient had a left-sided ventricular reentry (LV posterior wall) and a previous history of aortic coarctaction treated with aortic resection and graft positioning. In only one case (patient with left-sided ventricular reentry), activation mapping was not performed due to hemodynamically instability of the patient during tachycardia; in that case, the ablation of local abnormal ventricular activity (LAVA) and late potentials (LPs) during sinus rhythm was performed with non-inducibility of any ventricular arrhythmia as the ablation target.

Acute procedural success (tachycardia termination and/or non-inducibility) was reached in all patients without periprocedural complications. Only two patients underwent additional ablation lines during the same procedure due to the development of a different tachyarrhythmia during programmed stimulation and were no more inducible after further ablation. The mean ablation time was 24 ± 9 min (range 5–69 min).

### 3.3. Follow-Up

Six months after ablation, a transthoracic echocardiography exam was performed on all patients with a mean LVEF of 54 ± 9.2% (22–70%). After the ablation procedure, nineteen patients (95%) were under antiarrhythmic therapy (seventeen treated with beta-blockers and six with amiodarone). During a median follow-up period of 207 weeks (IQR 80 weeks), eight patients (40%) had arrhythmia recurrence, with a median time to recurrence of 41 weeks. Of these, five patients received ablation for atrial tachyarrhythmias (one with TGA with atrial switch, ASD, VSD, and dextrocardia; one with TGA corrected with arterial switch; one with ASD, and two with TOF) and three patients for ventricular tachycardia (two with TOF and one with corrected aortic coartaction). Five patients (25% of the total population) underwent a second mapping and ablation procedure that revealed the presence of a new reentrant circuit in all but one case, while one patient had a conduction gap across the previous ablation line. In all the patients undergoing a second procedure, there was no significant increment of the bipolar scar area size (36.3 ± 37.6 at first ablation vs. 38.3 ± 37.3 at repeat ablation, *p* = ns). One patient with TOF died due to heart failure exacerbated by pneumonia two years after the VT ablation procedure. One patient with aortic coarctation underwent epicardial ablation of the VT in another center (procedural data not available) and had further recurrencies thereafter; he eventually remained arrhythmia-free after further escalation of his antiarrhythmic treatment. The remaining patient had no further arrhythmic episodes after escalation of the drug therapy.

### 3.4. Predictors of Arrhythmia Recurrence

Therapy with beta-blockers (*p* = 0.04) and with diuretics (*p* = 0.021) was more frequent among patients with arrhythmia recurrence. 

The presence of a bipolar scar itself did not predict arrhythmia recurrence, since all the patients presented some degree of bipolar scar during the electroanatomical mapping (*p* = ns).

**Figure 2:**
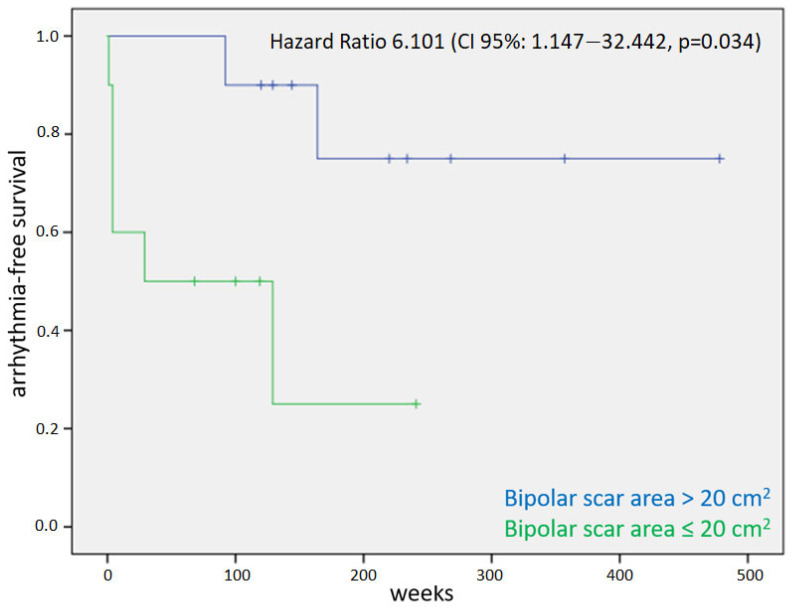
Kaplan–Meier curve for the primary endpoint of arrhythmic relapse in patients with and without an extension of bipolar scar >20 cm^2^.

The bipolar scar area >20 cm^2^ was identified as a possible cut-off value by means of the receiver operating characteristic (ROC) curve. At the Cox regression analysis, the extension of the bipolar scar area (hazard ratio 1.049, confidence interval 1.011–1.089, *p* = 0.011) and the presence of a bipolar scar area >20 cm^2^ (hazard ratio 6.101, confidence interval 1.147–32.442, *p* = 0.034) were identified as predictors of arrhythmia relapse. The results are summarized in Table 3. Figure 2 shows the relative Kaplan–Meier curve for the factor bipolar scar area > 20 cm^2^. 

## 4. Discussion

In our study, we investigated the role of the presence and extension of myocardial scars, assessed by electroanatomical bipolar mapping with multielectrode mapping catheters, in predicting arrhythmia relapse after the first ablation of atrial or ventricular arrhythmias in ACHD. The extension of the bipolar scar area and the presence of a bipolar scar area >20 cm^2^ were identified as predictors of arrhythmia relapse.

The total mapped area, LVEF, and ablation time did not predict the endpoint of the study. The assumption of diuretics or beta-blockers predicted a worse outcome, likely due to more severe heart failure causing further arrhythmic complications among patients taking these classes of drugs.

Structural remodeling caused by pressure and volume overload, along with surgical scars, leads to replacement and interstitial fibrosis, which represent critical areas of arrhythmogenesis among patients with CHD [20]. 

Late gadolinium enhancement (LGE) at cardiac magnetic resonance (CMR) is currently the gold standard for the assessment of ventricular fibrosis [21,22], but patients undergoing the ablation of ventricular arrhythmias often have implantable cardioverter defibrillators (ICDs), preventing accurate imaging because of artifacts. On the other hand, the imaging of atrial fibrosis by means of LGE at CMR remains controversial and is not widely adopted in clinical practice, mainly because of its suboptimal reproducibility [23,24]. 

Scar characterization by means of electroanatomical mapping is universally adopted in electrophysiology labs worldwide, and robust data have shown its usefulness in tissue characterization for both atrial and ventricular arrhythmic substrates [15,17,18,25]. To date, several studies have investigated the outcome and predictors of arrhythmia recurrence after catheter ablation among ACHD [7,9,11,12,13], but the role of myocardial fibrosis in this context has not been clearly demonstrated. 

All the patients in our study presented low-voltage areas during electroanatomical mapping. In the ACHD population, chamber dilatation with volume and/or pressure overload, and multiple previous surgical interventions, make the absence of myocardial fibrosis virtually impossible. In this population, low-voltage areas can be related to “natural” fibrosis, scars after cardiosurgery, or the presence of artificial materials. Hence, the presence of a bipolar scar itself could not predict arrhythmia recurrence. However, the extent of the bipolar scar predicted the study endpoint. In particular, we could recognize a bipolar scar cut-off of 20 cm^2^ to identify patients at a higher risk of arrhythmia recurrence. 

It is expected that the presence of extensive areas of scars in a cardiac chamber can give rise to multiple reentry circuits, sustaining different tachycardias. We performed the ablations during tachycardia (except in one case due to poor hemodynamic tolerance) and aimed at arrhythmia termination during radiofrequency delivery. After arrhythmia termination, we always performed programmed stimulation to assess the absence of the reinduction of the tachycardia. In one case, a different tachycardia was induced and ablated in the left atrium during the same procedure after ablation for a right-sided IART. In all the other cases, no arrhythmia was induced, and the procedure was considered completed. Eight patients (40%) had arrhythmia recurrence during the follow-up, in agreement with the previous literature data, which suggests a high recurrence rate in this population. Among the patients presenting arrhythmia recurrence who underwent a new catheter ablation, only one showed the same arrhythmia circuit during the second procedure. That means that, even after effective ablation of the responsible reentry circuit, the presence of extensive scars favors the creation of further potential arrhythmia circuits during the follow-up. Further chamber dilatation or enlargement of the scar area due to unfavorable hemodynamics might progressively change the conduction capability of the border zone until the conduction becomes as slow as needed to create a new critical isthmus that can sustain a reentry.

Based on our results, it may be postulated that, in ACHD presenting with large areas of scars during bipolar electroanatomical mapping, it can be worth spending more time, after ablation of the index tachycardia, to search for and eliminate any further potential critical isthmus, even in the absence of arrhythmia induction by programmed stimulation. This might theoretically reduce the rate of arrhythmia relapse. However, we did not perform any further ablation beyond the target arrhythmia during the index procedure, and hence, we cannot provide any recommendation in this direction. Further research is warranted in order to test this hypothesis, which is purely speculative. 

## 5. Study Limitations

The results of our study may be impaired by the small sample size; further studies on larger populations are needed to confirm our results. However, this was the first study to investigate the role of bipolar scars in predicting clinical outcomes among ACHD undergoing catheter ablation.

Our population was heterogeneous, including patients with different types of CHD, different surgical corrections, and patients with both atrial and ventricular tachycardias. However, few studies of the ablation of ACHD patients have reported large populations, because they represent a small subset of the total population of patients undergoing catheter ablation, and studies on homogeneous ACHD populations (e.g., patients with the same congenital defect undergoing ablation of the same arrhythmia) are hard to conduct due to enrollment difficulties.

## 6. Conclusions

ACHD presenting with atrial or ventricular arrhythmias show a good acute response to catheter ablation but a high rate of arrhythmia relapse during the follow-up. The extension of the bipolar scar area and the presence of a bipolar scar area >20 cm^2^ can predict arrhythmia relapse. Recurrent arrhythmias are often caused by circuits other than those previously ablated. The evidence of an extensive bipolar scar may identify a subset of ACHD patients at a particularly high risk of recurrence, thus suggesting the need for more extensive ablation aimed at eliminating all the potential future critical isthmuses. However, further studies on larger populations are warranted in order to confirm this hypothesis.

## Figures and Tables

**Table 1:** Structural heart disease, surgical correction, and clinical arrhythmia of the enrolled patients.

Pt	Age/Gender	Structural Heart Disease	Surgical Correction	Index Arrhythmia
1	64/M	ostium primum ASD, partial ACD	Surgical closure defect and mitral valve replacement	Left atrial reentrant tachycardia
2	52/M	TGA, ostium secundum ASD, VSD, dextrocardia	Mustard procedure (atrial switch)	Right atrial reentrant tachycardia
3	35/F	Ebstein’s anomaly	No surgical repair	Right atrial reentrant tachycardia
4	33/F	TGA	Rastelli procedure (arterial switch)	Right atrial reentrant tachycardia
5	48/F	TOF	Total correction	Left atrial reentrant tachycardia
6	50/F	ostium secundum ASD	Surgical closure defect	Right atrial reentrant tachycardia
7	31/M	ostium primum ASD, truncus arteriosus	Surgical closure defect and conduit-based RVOT reconstruction	Right atrial reentrant tachycardia
8	50/M	TOF	Total correction	Right atrial reentrant tachycardia
9	24/M	complete ACD, BAV, aortic coartaction	Surgical closure and valves replacement	Right atrial reentrant tachycardia
10	48/F	ostium secundum ASD, pulmonary valve stenosis	Surgical closure defect and transannular patch	Right atrial reentrant tachycardia
11	25/M	sinus venosus ASD	Surgical closure defect	Right atrial reentrant tachycardia
12	32 M	TOF	Total correction	Right atrial reentrant tachycardia
13	50/M	TOF	Total correction	Right ventricular reentrant tachycardia
14	38/M	TOF	Infundibular patch	Right ventricular reentrant tachycardia
15	32/M	TOF	Total correction	Right ventricular reentrant tachycardia
16	40/M	aortic coartaction	Resection of stenotic part and interposition of a graft	Left ventricular reentrant tachycardia
17	30/F	TOF	Total correction	Right ventricular focal extrasystole and tachycardia
18	30/M	TOF	Total correction	Right ventricular reentrant tachycardia
19	52/F	VSD, tricuspid valve atresia	Surgical closure defect and Fontan procedure	Right atrial reentrant tachycardia
20	27/M	TOF	Total correction	Right atrial reentrant tachycardia

ASD: atrial septum defect, ACD: atrioventricular canal defect, TGA: transposition of great arteries, VSD: ventricular septum defect, TOF: Tetralogy of Fallot, BAV: bicuspid aortic valve, RVOT: right ventricular outflow tract.

**Table 2:** Clinical characteristics of the study population.

	Total n of Patients, n = 20 (100%)	Patients without Relapse, n = 12 (60%)	Patients with Relapse, n = 8 (40%)	*p*-Value
Age (years)	39 ± 10	38 ± 12	42 ± 10	NS
Gender M	13 (65%)	6 (50%)	7 (88%)	NS
Heart rate (bpm)	83 ± 25	83 ± 25	83 ± 25	NS
LV EF (%)	53 ± 10	56 ± 11	48 ± 14	NS
Smoking	4 (20%)	2 (17%)	2 (25%)	NS
Familiar history of CVD	2 (10%)	1 (8%)	1 (13%)	NS
Hypertension	5 (25%)	3 (25%)	2 (25%)	NS
Obesity	3 (15%)	2 (17%)	1 (13%)	NS
Diabetes	0 (0%)	0 (0%)	0 (0%)	NS
Dyslipidemia	1 (5%)	1 (8%)	0 (0%)	NS
Beta-blockers	12 (60%)	5 (42%)	7 (88%)	0.04
ACEi	5 (25%)	3 (25%)	2 (25%)	NS
Calcium channel blockers	0 (0%)	0 (0%)	0 (0%)	NS
Diuretics	6 (30%)	2 (17%)	4 (50%)	0.021
MRA	3 (15%)	0 (0%)	3 (38%)	NS
Amiodarone	5 (25%)	2 (17%)	3 (38%)	NS
Ic antiarrhythmic agents	3 (15%)	2 (17%)	1 (13%)	NS
Sotalol	2 (10%)	2 (17%)	0 (0%)	NS
Antiplatelets	2 (10%)	0 (0%)	2 (25%)	NS
Anticoagulants	8 (40%)	6 (50%)	2 (25%)	NS
Digoxin	1 (5%)	1 (8%)	0 (0%)	NS

**Table 3:** Predictors of arrhythmia recurrence.

	Hazard Ratio	Confidence Interval 95%	*p*-Value
LVEF	0.962	0.909–1.1017	ns
Mapped area	1.002	0.998–1.1005	ns
Ablation time	1.004	0.952–1.058	ns
Bipolar scar area extension	1.049	1.011–1.089	0.011
Bipolar scar area > 20 cm^2^	6.101	1.147–32.442	0.034

## Data Availability

The data presented in this study are available on request from the corresponding author. The data are not publicly available due to privacy reasons.
